# Digital Health Program to Support Family Caregivers of Children Undergoing Growth Hormone Therapy: Qualitative Feasibility Study

**DOI:** 10.2196/55023

**Published:** 2025-02-05

**Authors:** Alba Jiménez-Díaz, Maitena Pierantonelli, Patricia Morte Coscolín, Amaia Salinas-Uhalte, Silvia Quer-Palomas, Octavio Rivera-Romero, Rocío Herrero, Luis Fernández-Luque, Rosa Baños, Ricardo C Berrios, Antonio de Arriba

**Affiliations:** 1 Departamento de Personalidad, Evaluación y Tratamientos Psicológicos Universitat de València Valencia Spain; 2 Unidad de Endocrinología Pediátrica Hospital Universitario Miguel Servet Zaragoza Spain; 3 Instituto de Investigación Sanitaria de Aragón Zaragoza Spain; 4 Adhera Health Inc. Santa Cruz, CA United States; 5 Instituto de Ingeniería Informática Universidad de Sevilla Seville Spain; 6 Electronic Technology Department Universidad de Sevilla Seville Spain; 7 Departamento de Psicología y Sociología Universidad de Zaragoza Teruel Spain; 8 CIBER of Physiopathology of Obesity and Nutrition (CIBEROBN) Madrid Spain

**Keywords:** growth hormone deficiency, mobile based solutions, caregivers, technology acceptance, digital health, children, therapy, feasibility study, health condition, psychological burden, quality of life, wellbeing, pediatric, mobile Health, mHealth, behavioral change, parent-child relationship

## Abstract

**Background:**

Caregivers of children with growth hormone deficiency often face emotional challenges (eg, stress) associated with their children’s health conditions. This psychological burden might affect children’s adherence to treatment and hinder their health-related quality of life (HrQoL). This assumption is leading to seriously considering multidimensional clinical approaches to pediatric health conditions where the emotional well-being of caregivers should be accounted for to optimize children’s health outcomes. Novel mobile health (mHealth) solutions based on emotional and behavioral change techniques can play a promising role because they are increasingly used within different health areas to support adaptive psychological functioning. However, whether and how mHealth solutions of this type of emotional well-being support caregivers of children with growth-related problems is an issue that needs to be clarified.

**Objective:**

This study aimed to gather qualitative information to better understand individualized experiences of caregiving of children undergoing growth hormone therapy (GHt) and perceived barriers or facilitators for the adoption of an mHealth solution called Adhera Caring Digital Program (ACDP).

**Methods:**

A total of 10 family caregivers were recruited at Miguel Servet Children’s Hospital, and they engaged with the ACDP for 1 month. The ACDP is a mobile-based digital intervention focused on promoting the overall well-being of family caregivers which provides access to personalized education, motivational mobile-based messages, and mental well-being exercises such as mindfulness or respiratory exercises. Subsequently, an individual semistructured interview was performed to gather qualitative user experience information.

**Results:**

The digital intervention was well-received. The ACDP was found to be useful, easy to use, and understandable, addressing all the difficulties expressed by caregivers. It was also noted to be particularly helpful at the beginning of the treatment and, for some families, became a natural tool that strengthened the parent-child relationship.

**Conclusions:**

The ACDP is a promising and well-accepted tool that enhances the experience of patients and caregivers. It improves the management of growth hormone deficiency and promotes the overall well-being of family caregivers.

**Trial Registration:**

ClinicalTrials NCT04812665; https://clinicaltrials.gov/study/NCT04812665

**International Registered Report Identifier (IRRID):**

RR2-10.1186/s12911-022-01935-1

## Introduction

Growth hormone deficiency (GHD) in infants is a treatable disease that causes short stature [1]. The most used growth hormone therapy (GHt) for the pediatric population is a daily injection of a recombinant human growth hormone (rhGH) [2]. The daily administration is performed out of the clinic and requires patients and caregivers to be active and engaged in the self-management of this health condition. This active self-management is of paramount importance because poor adherence to rhGH treatments can lead to reduced efficacy and increased health care costs [3]. However, as the rhGH treatment can last many years, self-management is challenging for patients and caregivers [4], and suboptimal adherence to the treatment has been constantly reported in the literature [5]. Several factors such as missed injections, poorer level of treatment understanding, discomfort with the injections, and misperceptions about the consequences of missed doses have been reported as potential causes of poor adherence [5].

Children with GHD often have to address other issues related to their short stature that impact negatively on their quality of life. As exemplified, Stephen et al [6] found that children with GHD had significantly worse quality of life and cognitive functions than children with normal stature. Varni et al [7] found that children with short stature, including those with GHD, reported statistically significantly worse fatigue than healthy children. Social withdrawal, shyness, anxiousness, and depression have also been reported as a consequence of GHD [8-15].

Caregivers play a key role in the management of the GHD. They are responsible for the treatment management and administration of GHt to children who are not autonomous enough. Furthermore, they have the responsibility of managing their children’s health condition, including all children’s quality of life issues. This role is not premeditated nor chosen, so caring could turn out to be burdensome and affect the caregiver both psychologically and physically [16]. For instance, stress was one of the reported consequences of caring for children living with GHD, presenting higher levels of stress among parents whose children were receiving GHt, but still had short stature [17]. This higher stress level may impact their environment, their health, and treatment adherence [16]. Therefore, caregivers are at risk of developing psychosocial problems, such as anxiety and depression, that could seriously impact the child’s health management. As an example, parental stress has been associated with poorer adherence of children to medical treatment [18]. Therefore, some authors have recommended assessing routinely caregivers’ stress and conducting psychosocial interventions aimed at promoting caregivers’ adaptation outcomes [17].

Currently, the health care sector is being transformed to benefit from the use of information and communication technologies. Digital health enables more accessible and potentially cost-effective alternatives to deliver family-centered interventions. Few studies have reported promising results on the efficacy of mobile health (mHealth)–based interventions for caregivers of children with chronic conditions [19]. Digital solutions, especially mobile apps, supporting patients and caregivers in the management of their disease have experienced significant and rapid growth. In GHD, Fernandez-Luque et al [20] found and analyzed 76 mHealth apps related to growth monitoring and growth hormone treatment available in the Android app store (Google). Most of these apps were intended for patients and caregivers. Some of the functionalities included in these self-management apps were education about GHD, education about growth tracking, and supporting and tracking adherence. However, the quality of digital health solutions is often not high enough and issues, such as trustworthiness or data privacy, are not appropriately addressed. This fact may lead to reduced adoption and engagement rates and, therefore, impact the effectiveness of the health interventions. In addition, the acceptability of digital health solutions by targeted users (patients or caregivers) is a key factor that also impacts the effectiveness of these interventions. Patients or caregivers will be reluctant to use digital health solutions that they do not find appropriate for them. There is still a need for conducting research on understanding the factors impacting caregivers’ adoption and acceptance of the use of mHealth apps supporting them in the management of pediatric diseases.

Several technology acceptance models and theories such as the Technology Acceptance Model (TAM) [21] or the Unified Theory of Acceptance and Use of Technology (UTAUT) [22] have been proposed in the literature. The UTAUT and its versions have been widely used in digital health [23-25]. The UTAUT proposes that 4 constructs play a significant role as direct determinants of user acceptance and usage behavior—performance expectancy, effort expectancy, social influence, and facilitating conditions. Performance expectancy is defined as “the degree to which an individual believes that using the system will help him or her to attain gains in job performance.” This construct is related to concepts such as perceived usefulness, extrinsic motivation, and outcome expectations. Effort expectancy is defined as “the degree of ease associated with the use of the system.” This construct is related to perceived ease of use. Social influence is defined as “the degree to which an individual perceives that important others believe he or she should use the new system.” This construct is related to subjective norms. Finally, facilitating conditions are defined as “the degree to which an individual believes that an organizational and technical infrastructure exists to support the use of the system.” In addition, the UTAUT defines 4 moderators (gender, age, voluntariness, and experience) that influence these determinants.

This research aims to gather qualitative information to better understand the psychological burdens experienced by caregivers of children undergoing growth hormone treatment, as well as the perceived barriers and facilitators related to accepting an mHealth solution that supports the self-management of GHD.

## Methods

### Recruitment

A total of 10 volunteer caregivers of children with GHD being treated by the physician AA from the Pediatric Endocrinology Unit at the Miguel Servet Children’s University Hospital were invited to participate during face-to-face hospital visits. The sampling was selected by convenience from June to July 2021, and none of them refused to join the study. To be included in the study, all the described criteria had to be met (Textbox 1).

**Inclusion and exclusion criteria.**

**Inclusion criteria**

- Adherence to growth hormone therapy (GHt) monitored in the last month before enrollment indicates a ratio of less than 85% (since it has been considered as an index of relatively suboptimal adherence) [26,27].
- Legal guardian of children who receive GHt in accordance with approved indications in Spain.
- Explicit agreement on data sharing regarding adherence to GHt gathered through Easypod Connect (a digital platform that monitors GHt).
- Participants must not report any limitations in the use of smartphones and smartphone apps.
- Participants must accept the terms of use and agree to install the Adhera Caring Digital Program mobile-based intervention app.
- Participants must sign the specific informed consent form for the study.

**Exclusion criteria**

- Candidates without an Android or Apple smartphone because the solution only works through these 2 operating systems.
- Reporting any limitation in the use of smartphone apps.
- Only 1 legal guardian per child can participate in the study.

### Procedures

At baseline, participants were requested to sign the informed consent and asked about demographic data and distress assessed by the Depression, Anxiety, and Stress Scale–21 (DASS-21) in its version in Spanish. After being introduced to the mHealth solution, they were guided to download, install, and configure the app on their own mobile phone. They were granted a free month of full access to the Adhera Caring Digital Program (ACDP).

After enrolling in the ACDP for 1 month, user experience was assessed with a semistructured interview. All the research was performed in Spanish.

### Ethical Considerations

The current research was approved by the Ethical Committee CEICA (Comité de Ética de la Investigación de la Comunidad de Aragón; number CP-CI PI20/494). All procedures followed were in accordance with the Helsinki Declaration of 1975, as revised in 2000. Participation in the study was voluntary and anonymous; no compensation was given. All participants gave their informed consent. Audio recordings were deleted once transcribed. All personal information was deidentified.

### Measures

#### DASS-21 Scale

The DASS-21 is a self-reported questionnaire divided into 3 scales that measure the emotional states of depression, anxiety, and stress. Each scale has 7 items, which are graded on a Likert scale from 0 to 3 (0: did not apply to me at all, 1: applied to me to some degree or some of the time, 2: applied to me to a considerable degree or a good part of the time, and 3: applied to me very much or most of the time). The scores are calculated by measuring the result of each scale multiplied by 2. The final result is classified as normal, mild, moderate, severe, or extremely severe [26,27].

#### Qualitative Interview

A semistructured interview was designed in order to gather the user experience after joining the ACDP for one month.

### Adhera Caring Digital Program

The ACDP is a mobile-based digital intervention focused on promoting the overall well-being of family caregivers which provides access to personalized education, motivational messages, and mental well-being exercises such as mindfulness or respiratory exercises. In this study, participants were invited to participate in the program for 4 weeks [28].

**Figure 1:**
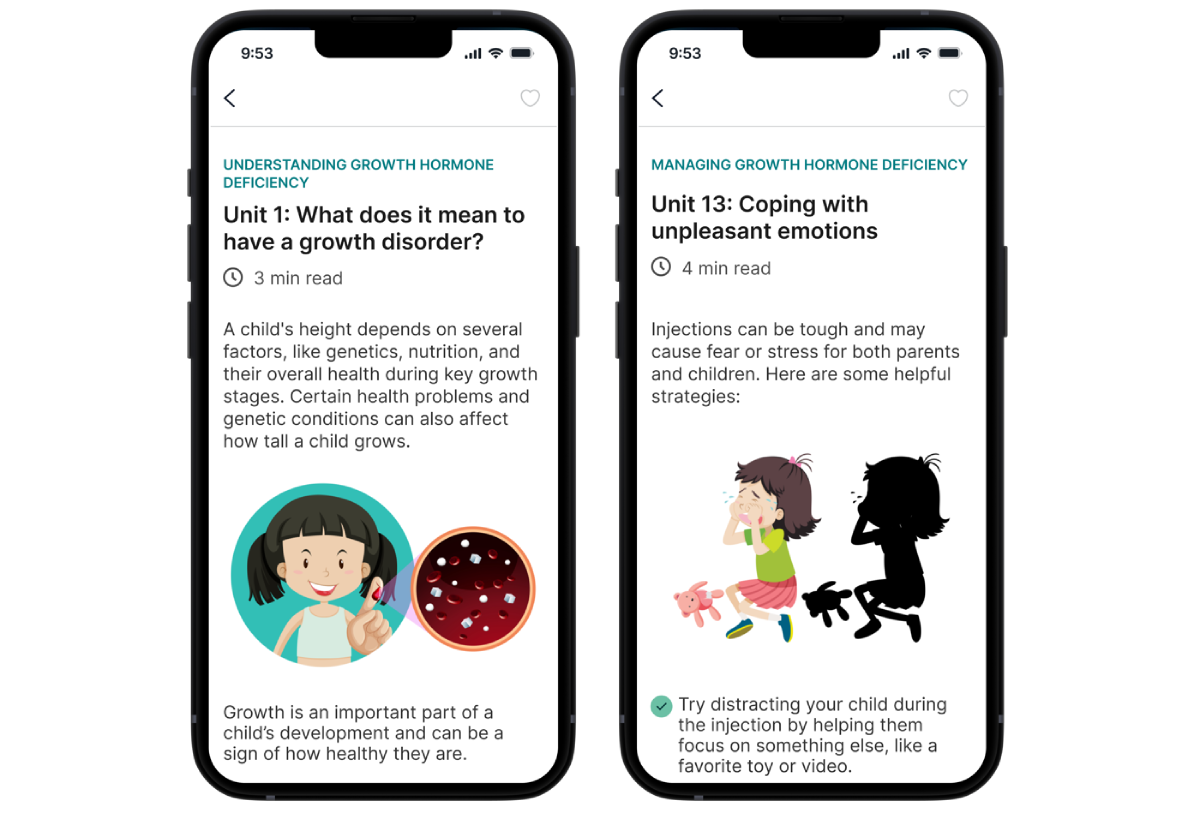
An Educational Unit within the app.

Psychoeducational modules provide educational content for parents or guardians of children with GHD (refer to Figure 1 for some examples). The contents include 39 units, classified into four sections: (1) managing GHD, (2) health habits to improve dealing with GHD, (3) adjusting to living with GHD, and (4) taking care of yourself to be able to take care of your child.

The program also includes a behavioral change module which complements and strengthens the knowledge of the psychoeducational module and provides lifestyle suggestions and action planning. It is done by delivering brief messages created by a multidisciplinary team of experts, including doctors and psychologists. The artificial intelligence–driven Adhera Precision Digital Companion Platform will select the tailored mobile-based messages to be sent (so the message selected will be personalized according to the patient profile, interests, and other peculiarities) [29]. This program incorporates the principles of personalized health education into a mobile platform, achieved by applying the Integrated Model of Behavioral Change [30] which is further expanded using recommender systems.

The ACDP is part of the Adhera Health Precision Digital Companion Platform, which has been developed using the best practices regarding data protection and quality management in accordance with the guidelines of ISO (International Organization for Standardization) 27001 and ISO 13465.

### Interview Data Collection and Analysis

Participants were individually interviewed by 2 trained doctors (physicians) from the hospital. The training was provided by the sponsor’s principal investigator through a workshop. The interviews were performed at the hospital or by secured video calls with caregivers or family and researchers being involved only. The mean duration of each interview was approximately 15 minutes. Individual interviews were anonymous, audio-recorded, manually transcribed, and anonymized by MP. MP and AJ-D managed and analyzed the transcripts using ATLAS.ti (Lumivero) Scientific Software Development GmbH (ATLAS.ti version 7.5.4 1993-2012 Windows). They performed an independent parallel analysis and arrived at the same conclusions regarding the themes that emerged during the interviews. Some translations of the interview can be consulted in Multimedia Appendix 1.

A data-driven inductive strategy was generally followed. However, some UTAUT model concepts [31] were considered regarding some aspects of technology acceptance, following a deductive-like approach in this specific case. The study followed the consensual qualitative research methodology [31,32]. In this approach, core ideas are identified and organized into categories, which are embedded in broader domains. To do so, 2 reviewers (MP and AJ) independently extracted and organized the data. They then compared their findings in a series of feedback meetings to ensure objectivity and reach interrater consensus. A third reviewer (RH) was consulted in case of discrepancy.

## Results

### Overview

The characteristics of the sample (n=10) are described in Table 1. The majority of participants were female (80%) with a mean age of 44.9 (SD 4.41) years. Most of the caregivers were married (70%). In terms of education, the majority held a university degree (70%). The children were primarily female (70%) with a mean age of 10.6 (SD 2.5) years. The average age at the start of was 5.9 (SD 1.66) years and had been under treatment for a mean of 60.3 (SD 23.59) months. The mean adherence rate was 73.44% (SD 28.6).

**Table 1:** Descriptive characteristics of the sample.

Characteristic			Statistical value (N=10)
**Caregiver’s sex, n (%)**			
	Male	2 (20)	
	Female	8 (80)	
Caregiver’s age, mean (SD)			44.9 (4.41)
**Caregiver’s marital status, n (%)**			
	Married	7 (70)	
	Divorced	3 (30)	
**Education, n (%)**			
	High school	2 (20)	
	Professional training	1 (10)	
	University degree	7 (70)	
**Child’s sex, n (%)**			
	Male	3 (30)	
	Female	7 (70)	
Child’s age, mean (SD)			10.6 (2.5)
Child’s age at the start of the treatment, mean (SD)			5.9 (1.66)
Time under treatment (months), mean (SD)			60.3 (23.59)
Adherence rate to growth hormone therapy (%), mean (SD)			73.44 (28.6)

### DASS-21 Scale

At baseline, DASS-21 showed that the majority of participants had no symptoms of depression (70%), anxiety (90%), or stress (70%). However, some of the participants had mild (20%) or moderate (10%) depression, severe anxiety (10%), and mild (20%) or severe (10%) stress, as described in Table 2.

**Table 2:** Depression, Anxiety, and Stress Scale–21 results on baseline.

		Participants, n (%)
**Depression**		
	Normal (0-4)	7 (70)
	Mild (5-6)	2 (20)
	Moderate (7-10)	1 (10)
	Severe (11-13)	0 (0)
	Extremely Severe (14+)	0 (0)
**Anxiety**		
	Normal (0-3)	9 (90)
	Mild (4-5)	0 (0)
	Moderate (6-7)	0 (0)
	Severe (8-9)	1 (10)
	Extremely severe (10+)	0 (0)
**Stress**		
	Normal (0-7)	7 (70)
	Mild (8-9)	2 (20)
	Moderate (10-12)	0 (0)
	Severe (13-16)	1 (10)
	Extremely severe (17+)	0 (0)

### Interview Results

**Figure 2:**
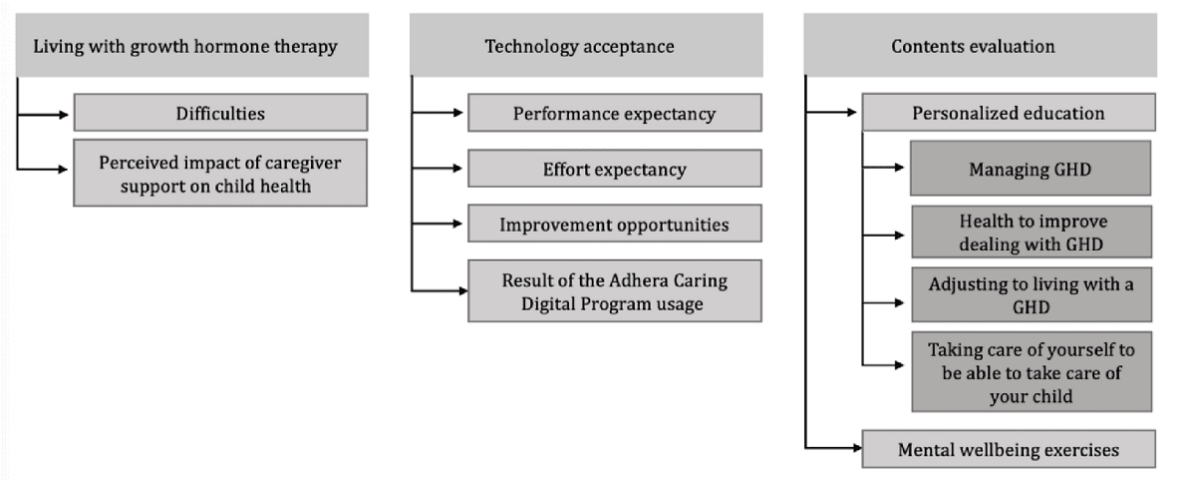
Categories or themes identified in the interviews. GHD: growth hormone deficiency.

A total of 3 domains were identified as shown in Figure 2, that are living with GHt, technology acceptance, and contents evaluation, containing a total of 8 categories. Furthermore, 1 category comprehended 4 subcategories.

#### Living With Growth Hormone Therapy

This domain encompasses the perceived impact of dealing with this condition on caregivers’ everyday lives. In total, 2 categories were highlighted, difficulties and perceived impact of caregiver support on child health.

#### Difficulties

This category was not included as a specific question of the semistructured interview. However, caregivers expressed concern about common issues. Beyond the diagnosis, participants reported children’s behavioral issues and individual characteristics that hinder the treatment administration. For instance, the child’s difficulties in understanding the disease promoted their refusal of hormone treatment.

She is a complicated child (…) that is my main problem. I do not know if it’s something to do with my daughter’s character or if I do not know how to deal with it.

Caregiver 06

The child said “why do I have to do this? I do not mind. I do not mind that people do not like it.”

Caregiver 09

Daily injections were reported to be difficult for both, children and caregivers. Participants also expressed great concern about their children’s lack of autonomy toward treatment. As children get older and socialize outside the nuclear family, the lack of autonomy in treatment becomes more evident, limiting children’s independence and increasing caregivers’ distress.

She will be thirteen this summer, fourteen next summer, and that has caused me a lot of problems. I can’t let her sleep anywhere, if I let her, I have to go and perform the injection. Wherever she is. This means that I can’t leave her with friends. She tells me, “I want to sleep over.” Well, no. If you don’t inject by yourself, you can’t stay. It is impossible to go camping. It is a medication that has to be in a refrigerator... that she does not inject herself. How are you going to send her? My daughter has never been to camp.”

Caregiver 02

Caregivers’ fear of hurting their children or mismanaging treatment was also raised.

I don’t know if there are many children who inject themselves. Maybe there are a lot, but I haven’t been able to. It’s impossible for him; he has needle phobia.

Caregiver 02

My mouth went dry every time I had to inject her.

Caregiver 02

#### Perceived Impact of Caregiver Support on Child Health

Participants were asked whether they felt that being supported could influence their children’s health. Many participants agreed that it was important to maintain a certain level of calmness and well-being as they could be perceived as role models by the children. Consequently, it is likely that if caregivers are angry or anxious these feelings would be transmitted to their children, increasing resistance to treatment.

If the parents feel bad, the children will feel awful (...). If I was stressed at the beginning, I transmitted the stress to the child, and it wasn’t good. The children see us as... well, “if mom says that everything is going to be fine, then it will be fine.” It is very important [to know] that if we are down, we transmit fear to the children. I think it is very important.

Caregiver 04

The importance of achieving personal well-being in order to effectively care for others was also emphasized.

It would be the main subject of the twenty-second century: taking care of yourself in order to take care of others.

Caregiver 09

#### Technology Acceptance

This domain highlights that, in general, caregivers expressed a positive attitude toward the use of technology as emotional and self-management support during the growth hormone treatment.

During the analysis, four categories concerning the usefulness of the digital solution were identified, of which the first 2 were directly related to the UTAUT model: (1) performance expectancy, (2) effort expectancy, (3) improvement opportunities, and (4) result of the ACDP usage.

#### Performance Expectancy

According to the authors, this construct refers to the user’s perception of how effectively the technology will help them achieve improvement [31]. In this study, performance expectancy refers to the caregiver’s perception of the ACDP’s usefulness, regarding emotional support and management of the GHt. Overall, the participants agreed with the idea that digital solutions can be a useful and positive means of supporting the role of caregivers. They often mentioned that the wide variety of resources present in the ACDP allows very different types of families enjoy appropriate support for their specific needs. For instance, some caregivers stated how the ACDP helped them normalize the GHD and explain it openly to their children while paying attention to maintaining children’s self-esteem.

Overall, I found it very useful, not only the theoretical part but the messages that appear on your cell phone. …. It is not that I see a part that is not useful and another that is more useful. I understand that for each person there may be a part that is more useful than others... But in general, I would give it a 9… (out of 10).

Caregiver 01

I really liked it because, of course, I didn’t know how to encourage my girl and how to explain to her that this is something normal.

Caregiver 04

In addition, the ubiquity of the resources in the ACDP, available anywhere and anytime, was also appreciated. This, together with the fact that they perceived the contents of this digital solution to be highly reliable and clinically validated, made the caregivers feel reassured.

It is good to include explanations because sometimes in the consultation you feel overwhelmed and end up having doubts. Then the application reminds you everything again.

Caregiver 03

Well, what I have seen is that this is the information that should be there. ... In a consultation with the doctor, he will explain the medical issues. Then, on your own, you will go to Google and you will also search for medical issues. You will find good, bad, and irrelevant information. In addition, the information might be useful for you or not, and you might rely on information unrelated to your case. That is due to how Google works and how a low-skilled person might interpret the information. Regarding the emotional side, I do consider that it would be a very good path, a very good course or training, to have it at the beginning because it has the information that needs to be there. For me, this is the starting point. From there, you can find more, but there's the information you need to have.

Caregiver 09

Nonetheless, one parent expressed that they preferred receiving face-to-face support rather than having a mobile app for this purpose. Also, they shared that their child did not have some of the behavioral issues mentioned in the program. Thus, the caregiver stated that they could not apply all the information received within the digital program to make the child understand the need for treatment, but they saw it as useful to support parents.

An application, no. A child psychologist, yes. A face-to-face, yes. I don’t think the app would be useful [to make the child understand the need for treatment](...) Also, because of treatment insecurities, it would be useful for supporting parents, but through an app, I don’t think it would work one hundred percent (...) I think that, apart from an application, a set [of activities] as well. A talk with parents...

Caregiver 08

Another person stated that one of the strategies proposed for managing the app was not suitable for their children because of their age. However, the caregiver expressed their will to continue trying other strategies suggested within the digital intervention.

And, in fact, I read it again and told her, “well, I’m going to try again and we’re going to try to do it like this.” Also, XXX is already 7 years old, so distracting her is complicated … no matter how much you form the habit: let’s get her involved, let’s play music, let’s do it in some other way. She realizes it... You cannot fool her anymore. She knows that, in the end, [there is] an injection that she does not want. However, I will try these guidelines and ideas again.

Caregiver 06

#### Effort Expectancy

The effort expectancy concept refers to how easy the users perceive it will be to use a technology or system [31]. In this study, this construct is expressed in terms of the user experience and user interface, the structure of the information, and the degree of understandability.

The ACDP was described as easy to use by 8 participants. The adjectives “intuitive” and “beautiful” were also mentioned by some participants. It was stated that the user experience allows a peaceful state of mind which in turn facilitates the comprehension of its contents. The navigation flow was also appreciated.

I did not find it difficult. I found it, on the opposite side, very simple, very comfortable, very pleasant. Even the presentation of the application. Or I don’t know what to call it, by folders and then more units. I find it very comfortable. I find it very nice. They are calm, it helps to make it more relaxed reading, which is what is needed for this type of information. It seems to me that you have taken great care of that.

Caregiver 07

However, 1 participant reported that she found the app neither pleasant nor unpleasant but contextualizing that was not important for her.

Neither particularly attractive nor obviously unpleasant [the application]. No, the design maybe... Because I pay little attention to those things and more to the information.

Caregiver 01

Most of the participants did not encounter technical issues while using the app. However, 3 users reported that they could not access a specific kind of notification related to motivational mobile-based messages. Another participant reported having issues accessing but it was smoothly solved through a password recovery process.

Regarding the understandability of the contents within the ACDP, most of the users described them as easily understandable, as the contents were described in a clear manner, using precise, direct, and natural language.

It seemed to me a very direct and very clear form of expression. It is very clear that anyone can understand it. That is very important. I found the information very clear. No difficult words or expressions that are difficult to understand. No, on the contrary, it seems to me that it is very well written. For easy understanding, yes. … They are like very short units that make you think. It doesn’t make you read everything at once. It makes you think, and it is very good.

Caregiver 07

Nonetheless, a nonnative Spanish speaker reported that they would find some difficulties related to technical vocabulary related to the medical condition. These difficulties were solved by searching the words in a dictionary.

Finally, most of the users reported that the content was well-organized in diverse units, and provided brief pieces of information, with an interface similar to that of the social network platforms they are used to. This amount of information allowed for a better acquisition of knowledge and deeper reflections, compared with the relative overload of information that they felt during the diagnostic clinical visit.

The simplicity and clarity, the information come in very concise pills. It does not require a super long text that can make you feel tired, the information is very well dosed. … It is distributed in very chewable doses. In the open world we live in, we are more and more used to the Twitter context, with just a few characters. So, I think that this information and training is quite well dosed.

Caregiver 01

#### Improvement Opportunities

This category reflects that there is always room for improvement. The main limitation of the digital program was the moment in the patient journey when the digital program was introduced, which was mentioned by 7 participants. They declared that the digital intervention would have been especially useful right after the initial diagnosis, notwithstanding it still adds value to the GHD management at the present time.

I think I told you that at the beginning. I think it is a very positive, very good application. I think it will help families who are just starting out a lot, it will clarify things for them. And for families that have been using it for a long time, if it is shared with them, it will also strengthen what we have been doing for years. For those who are just starting out, it will really help them a lot more. As I was reading it I was saying “I wish I had had this when I started with this.”

Caregiver 07

The participants also commented on some desired additional content for the program. The parents mentioned that including techniques to involve the grandparents in the treatment (when possible) would be of interest. Also, a parent proposed including storytelling to reflect the reality of living with GHD. Thus, stories could be read before the treatment application, to help the child understand and normalize both the condition and its treatment.

I would look for more alternatives for the parents. It’s very good to award prizes, it’s very good to motivate them. But the motivation often also comes from the fact that there are alternatives for the children. I don’t know [motivation] for them to start self-injecting. I don’t know whether to look for other alternatives, other formulas, other... I don’t know. Because I am looking for it, I told him, “be aware that if I inject you, you won’t be able to stay anywhere. If I inject you, you won’t be able to go camping.” I don’t know, look for some... some story motivating to [complete] a process, as ... the stories for children in the autism spectrum regarding how to go to a birthday, how to behave or socialize. I don’t know if there is something uncomfortable like an injection, and there is some story or something that can be read to the children during nights that they can motivate themselves with. I don’t know if there are any stories on the market. There surely will be.

Caregiver 02

Furthermore, some parents suggested that they would appreciate having a section with testimonials of other families living with GHD so that they could learn from others’ experiences, or even get in touch with other families. Finally, a participant stressed the task of having a larger number of mindfulness and breathing exercises.

I don’t think anything should be removed. Add what I was telling you, apart from those two small meditation and breathing practices. If there were any more, it would be good. I don’t know if there could be some kind of sharing of experiences among people who are among parents, among children. Surely there are resources that we can use with each other... or situations how to handle them. I don’t know if it is feasible in the application or not.

Caregiver 06

#### Result of the Adhera Caring Digital Program Usage

All the core ideas regarding the benefits of using the program fall within this category. Participants agreed with several benefits that the ACDP had provided them. On the one hand, it allows the normalization of their children’s condition. This generates, for instance, increased self-esteem and decreased embarrassment in children. On the other hand, parents indicated that using the app leads to clarification of general concepts and knowledge about the disease, which empowers them. Finally, in the case of joint use with their children, the improvement in the relationship between caregivers and children was highlighted. The above-mentioned practical exercises have been described as “their moment together,” which would be missed after the study was completed.

It’s a perfectly normal thing, it’s not like this happened to my daughter and she was born like this. It’s normal. It’s a disease like any other that can be cured. … she was a little embarrassed and [after] reading that with me... well, “look, I shouldn’t be ashamed, that’s the way it is. On the contrary, I have to say that I am brave, look. That I inject myself. And I’m going to grow up, and there’s no problem.” … I think I get along with her much better than before. It gives her more confidence, I don’t know how to explain it. We accept it more.

Caregiver 04

#### Contents Evaluation

This domain encompasses the evaluation of the specific contents included in 2 components of the ACDP, personalized educational and mental well-being exercises.

#### Personalized Education

This category was organized in subcategories regarding the 4 sections of the personalized education section of the ACDP.

##### A. Managing GHD

In this section of the program, caregivers could obtain general information and advice on treatment management. Although it has been noted that this could be redundant as it is an explanation that the family receives when the patient is diagnosed, most participants agreed that receiving accurate and clear information resulted in a great benefit. Specifically, a sense of increased self-confidence and acceptance was highlighted in both patients and caregivers.

This information is missing at the beginning and creates uncertainty. Thinking about what we put in it and what effects it will have.

Caregiver 08

Among the most frequent core ideas were advice on how to mitigate the pain of the injection, or the reward system to promote treatment self-management.

(Talking about benefits of using the solution). Using ice or talking about something else... the typical advice given to mitigate the pain perception due to injections or the information given. For example, I read that sometimes it causes pain and other times it does not. It is something that we have experienced, and it is not known why. To me, it is something that catches my attention. I never knew if it was because the child was complaining because it really hurts, or because the needle is really very fine. So, I think having that knowledge regarding sometimes it hurts but you don’t know why and sometimes it doesn’t hurt is really important.

Caregiver 07

##### B. Health Habits to Improve Dealing With GHD

Some units of the personalized education section were aimed at promoting healthy habits and, more specifically, enhancing healthier eating and sleeping habits. Overall, these contents were labeled as “interesting” and “important.” However, 3 participants reported that they were already aware of that information since it had been provided by schools and pediatric health care providers. To them, this information was not that necessary.

Well, less useful, but not because they are not useful. Perhaps, for example, the component focused on healthy eating. It is very general information, but remembering it is important because not all families have the same opinion regarding diet or the importance of healthy eating. But maybe that’s the less useful part if I have to say one.

Caregiver 07

##### C. Adjusting to Living With a GHD

Considering the emotional impact of the diagnosis on the children’s mental health, the program proposed the necessity of providing clear information about emotions and advice on how to strengthen the bond with the caregivers. Participants evaluated positively this content, being a topic that is rarely addressed by medical staff at diagnosis.

In my case, the first section related to the disorder. I can remember things that perhaps would have been forgotten.

Caregiver 06

##### D. Taking Care of Yourself to Be Able to Take Care of Your Child

In line with the aforementioned, it is also important to consider the circular process of caring for oneself in order to care for others. Therefore, more content concerning the emotional management of caregivers was also suggested. They acknowledged the importance of their own emotional issues in the development of their children’s treatment.

#### Mental Well-Being Exercises

These exercises included visually guided mindfulness and breathing exercises. This category gathers the users’ opinions regarding mental well-being exercises. A caregiver described that practicing these exercises before applying the treatment had strengthened their relationship.

... I think I get along with her much better than before ... “mom, look what I can do.” It gives her more confidence, I don’t know how to explain it. ... It’s our moment. She even told me the day before yesterday “when I don’t do the sessions anymore it will seem strange to me, mom. That was our moment” I think it affects a lot, we talk about many things, and since we read it together (talking about the educational content) ... we commented on it.

Caregiver 04

Some caregivers expressed their curiosity toward these practical exercises and found them appealing and enjoyable. Only 1 caregiver reported that they did not receive any benefit from these exercises, which might be related to their specific preferences as stated.

I especially liked the meditation practice. And I would tell you, I would include a little bit more. Regarding caregivers, I liked all contents focused on working as a support, as a caregiver.

Caregiver 03

## Discussion

### Principal Findings

Caregivers of children undergoing GHt have reported several factors such as difficulties in understanding the disease or fear of hurting their children that may significantly impact the disease management. The role of caregivers is crucial in the management of GHD, and they have reported their well-being as a very important factor in being able to care for their children. Indeed, our results show that some caregivers have symptoms of depression, anxiety, or stress. In these circumstances, digital health enables the provision of digital services and tools supporting them in the care of their well-being and in the management of their children’s disease. In this study, caregivers have expressed a positive attitude toward the use of mHealth solutions for health and well-being management. This finding is in line with those reported by other authors [32]. The results of this study support the ACDP as a feasible and potentially effective tool for caregivers of children undergoing treatment for GHD.

Caregivers’ empowerment plays a key role in the effective management of GHD. Unfortunately, during routine consultations, it is not always feasible to cover all the aspects and doubts related to chronic diseases [32], especially the mental well-being of the caregiver. Digital health allows the provision of educational materials that are available just in time. Educational content must be designed not only to cover any lack of knowledge but also to encourage, empower, and optimize the caregivers’ role as a manager of the GHD. In this study, caregivers realized and expressed the importance of emotional well-being as well as healthy habits and adequate control of unpleasant situations related to their child’s condition. Considering that treatment adherence and the patient’s health can be influenced by the caregiver’s mental health [17], the ACDP can be a valuable part of the caregiver and patient journey. It is especially relevant once some participants expressed that the subcategory “family adjusting to living with a GHD” is not a common subject addressed during the diagnosis consultations. In this sense, participants in this study considered important not only the educational content but also the dispensing time of the digital health solution. Caregivers consider that the digital program is so useful that should be offered right after the diagnosis of the disease. To maximize their usefulness, digital health programs focused on helping caregivers manage their mental well-being and the day-to-day of their children’s disease should be offered at the right time. Furthermore, it was suggested to include a testimonials section or a virtual space where families in the same situation can be in touch. These social features may impact user’s motivation resulting in an increased adoption rate. This finding reinforces the fact that digital health can offer effective and motivational services for caregivers’ unmet needs that complement clinical practice [33,34].

Regarding the technology acceptance defined by the UTAUT theory [22-25], ACDP has been perceived positively by caregivers on performance expectancy and effort expectancy. The most important points of digital health solutions are that they can be used autonomously in the natural environment of the user, so usefulness, ease of use, and likeability are key [35,36]. The results indicate that the program seems to adjust to the core aspects of digital solutions as well as to cover all the needs and difficulties of participant caregivers of children and the user experience leads to a peaceful state of mind. Caregivers’ stress levels can affect treatment adherence and the child’s health [16], so giving them different tools to manage the disease seems to be a cardinal element in improving the quality of life of both. Therefore, understanding the factors that influence caregivers’ adoption and acceptance of mHealth apps is essential for the development of effective digital health interventions [20]. Our study contributes to this understanding, addressing the barriers to acceptance, and adoption among caregivers managing pediatric diseases, including data security.

An interesting result is that the program has become a familiar intervention even though its target is caregivers. Some parents used mental well-being exercises together with their children, which has been reported as a great tool to improve the relationship between the caregiver and the child. Further research with higher samples will be conducted to explore deeply this promising result and analyze how this family intervention could impact caregivers’ motivation, engagement, and well-being.

### Limitations

This is a local study located in Zaragoza (Spain) with a small sample of 10 caregivers. Because of inclusion and exclusion criteria, people with low digital literacy were not able to participate. Interviewers were not experts but received training for the purpose of the study. Besides, although the general prevalence of GHD is higher in boys, most caregivers participating in this study were female with daughters affected with GHD, thus, parents with GHD sons might be underrepresented as well as male caregivers. Finally, the program needs further study to include recently diagnosed children.

Although there are quantitative questionnaires to measure the acceptability of digital solutions [37,38], we opted for a qualitative approach in order to get a more comprehensive understanding of the psychological burden experienced by caregivers of children undergoing growth hormone treatment, as well on the factors that influence the acceptability of the digital solution. Due to the post-positivist nature of the consensual qualitative research methodology, an interjudge reliability index is not calculated. However, trustworthiness in the qualitative analysis is guaranteed through iterative discussions between reviewers to share their independent interpretation of the qualitative data. Where consensus is not achieved, a deeper discussion is held until consensus is reached or a third independent reviewer is consulted.

### Conclusions

In conclusion, the ACDP shows good acceptance results for family caregivers of children undergoing GHt. The interviews helped identify aspects for further refinement and improvement of the program, including a more intensive focus on the communication between parents and children. This study provides insights into how digital interventions can better support families of children undergoing growth hormone treatment.

## Appendix

Multimedia Appendix 1 Quotes from participants.

## Abbreviations

ACDP: Adhera Caring Digital Program
DASS-21: Depression, Anxiety and Stress Scale–21
GHD: growth hormone deficiency
GHt: growth hormone therapy
HrQoL: health-related quality of life
ISO: International Organization for Standardization
mHealth: mobile health
rhGH: recombinant human growth hormone
TAM: Technology Acceptance Model
UTAUT: Unified Theory of Acceptance and Use of Technology

## Data Availability

The datasets generated during and/or analyzed during this study are available from the corresponding author on reasonable request.
