# Correction: Digital Health Program to Support Family Caregivers of Children Undergoing Growth Hormone Therapy: Qualitative Feasibility Study

**DOI:** 10.2196/76508

**Published:** 2025-07-18

**Authors:** Alba Jiménez-Díaz, Maitena Pierantonelli, Patricia Morte Coscolín, Amaia Salinas-Uhalte, Silvia Quer-Palomas, Octavio Rivera-Romero, Rocío Herrero, Luis Fernández-Luque, Rosa Baños, Ricardo C Berrios, Antonio de Arriba Muñoz

**Affiliations:** 1 Departamento de Personalidad, Evaluación y Tratamientos Psicológicos, Universitat de València Valencia Spain; 2 Unidad de Endocrinología Pediátrica Hospital Universitario Miguel Servet Zaragoza Spain; 3 Instituto de Investigación Sanitaria de Aragón Zaragoza Spain; 4 Adhera Health Inc Santa cruz, CA United States; 5 Instituto de Ingeniería Informática, Universidad de Sevilla Sevilla Spain; 6 Electronic Technology Department, Universidad de Sevilla Sevilla Spain; 7 Departamento de Psicología y Sociología, Universidad de Zaragoza Teruel Spain; 8 CIBER of Physiopathology of Obesity and Nutrition (CIBEROBN) Madrid Spain

In “Digital Health Program to Support Family Caregivers of Children Undergoing Growth Hormone Therapy: Qualitative Feasibility Study” (JMIR Pediatr Parent 2025;8:e55023) the authors made one correction.

The following affiliation was added to authors RH and RB:

CIBER of Physiopathology of Obesity and Nutrition (CIBEROBN), Madrid, Spain

The correction will appear in the online version of the paper on the JMIR Publications website together with the publication of this correction notice. Because this was made after submission to PubMed, PubMed Central, and other full-text repositories, the corrected article has also been resubmitted to those repositories.

