# Supplementary material for: Digital Health Program to Support Family Caregivers of Children Undergoing Growth Hormone Therapy: Qualitative Feasibility Study
Source: JMIR Pediatr Parent. 2025 Feb 5;8:e55023. doi: 10.2196/55023 (PMC11840389; doi:10.2196/55023)
Supplement: Multimedia Appendix 1 [file pediatrics_v8i1e55023_app1.docx]

## Appendix I – Table with used quotes in Spanish and English

| Quotes | | |
| --- | --- | --- |
| Interview results section | **Spanish** | **English** |
| Difficulties | “Es una niña complicada (...) ese es mi principal problema de no saber cómo. No sé cómo explicarte. Yo ya no sé si es una cosa de carácter de mi hija o que yo no sé abordar el tema.  (Caregiver 06) | "She is a complicated child (..) that is my main problem. I do not know if it’s something to do with my daughter’s character or if I do not know how to deal with it.”  (Caregiver 06) |
|  | “Porque claro la niña decía ´y porqué me tengo que hacer esto, que a mí me da igual. Me da igual que no le guste a la gente y tal´"  (Caregiver 09) | “The child said ‘why do I have to do this? I do not mind. I do not mind that people do not like it’”. (Caregiver 09) |
|  | “No sé si hay muchos niños que se inyectan solos. A lo mejor hay un montón, pero yo no he conseguido. Se le hace imposible, le entra fobia al pinchazo”  (Caregiver 02) | “I don't know if there are many children who inject themselves. Maybe there are a lot, but I haven't been able to. It's impossible for him; he has needle phobia.”  (Caregiver 02) |
|  | “Va a cumplir trece años este verano, catorce el verano que viene, y eso me ha conllevado muchos problemas. Que no puedo dejarla dormir en ningún sitio, que si la dejo dormir tengo que ir antes a pincharla y marcharme. Este donde este. Esto conlleva que con amigos no la puedo dejar. Me dice “me quiero quedar a dormir” pues no. Si no te pinchas sola, no te puedes quedar. Ir de campamento imposible. Es una medicación que tiene que estar en una nevera, es una medicación que ella misma no se inyecta. Cómo la vas a mandar. Mi hija de campamentos no ha ido”  (Caregiver 02) | “She will be thirteen this summer, fourteen next summer, and that has caused me a lot of problems. I can't let her sleep anywhere, if I let her, I have to go and perform the injection. Wherever she is. This means that I can't leave her with friends. She tells me, "I want to sleep over." Well, no. If you don't inject by yourself, you can't stay. It is impossible to go camping. It is a medication that has to be in a refrigerator... that she does not inject herself. How are you going to send her?. My daughter has never been to camp.”  (Caregiver 02) |
|  | “Se me secaba la boca cada vez que le hacia el pinchazo”  (Caregiver 04) | “My mouth went dry every time I had to inject her” (Caregiver 04) |
| Perceived impact of caregiver support on child health. | “Si los padres están mal, los niños estarán fatal (…). Si yo estaba estresada al principio le transmitía a la niña el estrés y estaba mal. Los niños ven en nosotros como que… pues “si mamá dice que va a estar todo muy bien, pues va a estar bien”. Es muy importante que si nosotros estamos de bajón pues le transmitimos a los niños el miedo. Yo creo que es muy importante.”  (Caregiver 04) | “If the parents feel bad, the children will feel awful (...). If I was stressed at the beginning, I transmitted the stress to the child, and it wasn’t good. The children see us as... well, "if mom says that everything is going to be fine, then it will be fine". It is very important [to know] that if we are down, we transmit fear to the children. I think it is very important.”  (Caregiver 04) |
|  | “Sería la asignatura del siglo veintidós: el cuidarte a ti mismo para poder cuidar a los demás.”  (Cargiver 09) | “It would be the main subject of the twenty-second century: taking care of yourself in order to take care of others.”  (Caregiver 09) |
| Performance Expectancy | [Q17] En conjunto me ha parecido muy útil porque no solo la parte teórica, sino luego los mensajes que te van apareciendo en el móvil. Las cosas en general. No es que vea una parte que no sea útil y otra que sí que sea más. Entiendo que para cada persona pueda tener una parte más útil y otra no tanto. Pero el conjunto, en general le daría un 9. Y entiendo que eso, que habrá gente que alguna parte no le dé en alguna actividad, otras personas en alguna parte le dan una información, una utilidad bastante grande. Así que en conjunto yo le daría un 9 (de 10).  (Caregiver 01) | “Overall, I found it very useful, not only the theoretical part but the messages that appear on your cell phone. …. It is not that I see a part that is not useful and another that is more useful. I understand that for each person there may be a part that is more useful than others... But in general, I would give it a 9… (out of 10).”  (Caregiver 01) |
|  | A mí me ha gustado mucho porque claro, es que no sabía cómo animar a la chica y cómo explicarle que es algo normal.  (Caregiver 04) | “I really liked it because, of course, I didn’t know how to encourage my girl and how to explain to her that this is something normal.” (Caregiver 04) |
|  | Esta bien explicar porque a veces en la consulta te quedas con dudas. Entonces la aplicación te lo vuelve a recordar todo bien.  (Caregiver 03) | “It is good to include explanations because sometimes in the consultation you feel overwhelmed and end up having doubts. Then the application reminds you everything again.” (Caregiver 03) |
|  | Bueno, yo ahí lo que he visto es que esta la información que debe estar. Por decirlo de alguna manera. En una consulta con el medico te explicara las cuestiones médicas. Luego tu por tu cuenta te vas a meter en Google y vas a buscar también, yo creo que tema médico. Vas a encontrar cosas buenas, malas, regulares que te aplicaran, que no te aplicaran. Que te las tomaras como que te van a aplicar, pero en realidad no te aplican. O sea, lo que tiene el Google y la interpretación sin conocimiento por parte del que la recibe. Luego el tema emocional, sí que considero que era sería una muy buena vía, muy buen curso o formación, para tenerlo al principio porque ahí está la información que tiene que estar. Para mí, ni más información… o sea, el punto de partida de la información. A partir de ahí, puedes encontrar más información. Pero ahí está la información que tienes que tener.  (Caregiver 09) | “Well, what I have seen is that this is the information that should be there. ... In a consultation with the doctor, he will explain the medical issues. Then, on your own, you will go to Google and you will also search for medical issues. You will find good, bad, and irrelevant information. In addition, the information might be useful for you or not, and you might rely on information unrelated to your case. That is due to how Google works and how a low-skilled person might interpret the information. Regarding the emotional side, I do consider that it would be a very good path, a very good course or training, to have it at the beginning because it has the information that needs to be there. For me, this is the starting point. From there, you can find more, but there's the information you need to have.” (Caregiver 09) |
|  | Una aplicación no. Un psicólogo de niños, sí. Un cara a cara, sí. La aplicación no creo que le sirva (…) Por inseguridades del tratamiento también, pero a través de una aplicación tampoco lo veo bien (…) A través de una aplicación no creo que funcione a cien por cien. Creo que, aparte de una aplicación, un conjunto también. Una charla con padres, o no se… una vez cada (ininteligible) también.  (Caregiver 08) | “An application, no. A child psychologist, yes. A face-to-face, yes. I don't think the app would be useful [to make the child understand the need for treatment](...) Also, because of treatment insecurities, it would be useful for supporting parents, but through an app, I don't think it would work one hundred percent (...) I think that, apart from an application, a set [of activities] as well. A talk with parents...” (Caregiver 08) |
|  | Y de hecho vuelvo otra vez a leerlo y a decirle “bueno, voy a volver a intentarlo y vamos a volver a intentarlo hacer así”. También, XXX ya tiene 7 años, entonces hay cosas que ya la engañas entre comillas. Como decirte, por mucho que tú creas el hábito: vamos a hacer partícipe, vamos a poner música, vamos a hacerlo de alguna otra manera. Ella ya llega… lo veo más casi… bueno, no lo sé si más para pequeños o con otro carácter que XXX. Ya XXX no la engañas. Tú cuéntame toda la historia que quieras, pero al final va a acabar en un pinchazo que yo no quiero que me des. Pero claro que sí, me parecen pautas e ideas las cuales voy a volver a intentar y retomar.  (Caregiver 06) | “And, in fact, I read it again and told her, "well, I'm going to try again and we're going to try to do it like this". Also, XXX is already 7 years old, so distracting her is complicated … no matter how much you form the habit: let’s get her involved, let’s play music, let’s do it in some other way. She realizes it... You cannot fool her anymore. She knows that, in the end, [there is] an injection that she does not want. However, I will try these guidelines and ideas again.” (Caregiver 06) |
| Effort Expectancy | Difícil no la he encontrado. La he encontrado, al contrario, muy sencillo, muy cómodo, muy agradable. Incluso la presentación de la aplicación. O, no sé cómo llamarle, por carpetas y luego más unidades. Me parece muy cómoda. Me parece muy agradable. Son tranquilos, ayuda a que sea una lectura más relajada que es lo que se necesita para este tipo de información. Eso me parece que lo habéis cuidado mucho.  (Caregiver 07) | “I did not find it difficult. I found it, on the opposite side, very simple, very comfortable, very pleasant. Even the presentation of the application. Or, I don’t know what to call it, by folders and then more units. I find it very comfortable. I find it very nice. They are calm, it helps to make it a more relaxed reading, which is what is needed for this type of information. It seems to me that you have taken great care of that.” (Caregiver 07) |
|  | Ni especialmente atractiva ni tampoco obviamente desagradable [la aplicación]. No, el diseño quizás… Porque me fijo poco en esas cosas y más en la información.  (Caregiver 01) | “Neither particularly attractive nor obviously unpleasant [the application]. No, the design maybe... Because I pay little attention to those things and more to the information.” (Caregiver 01) |
|  | Me ha parecido una forma de expresión muy directa y muy clara. Muy que cualquier persona lo puede comprender. Eso es muy importante, me ha parecido una información muy clara. Sin palabras de difíciles o expresiones difíciles de comprender. No, al contrario, me parece que está muy bien redactada. Para una fácil comprensión, si.  (Caregiver 07) | “It seemed to me a very direct and very clear form of expression. It is very clear that anyone can understand it. That is very important, I found the information very clear. No difficult words or expressions that are difficult to understand. No, on the contrary, it seems to me that it is very well written. For easy understanding, yes. … They are like very short units that make you think. It doesn’t make you read everything at once. It makes you think and it is very good”  (caregiver 07) |
|  | la sencillez y la claridad y que la información viene en píldoras muy concisas. Que no se requiere un texto súper largo que te puede dar pereza, sino que esta muy bien dosificada la información… Que se distribuya en dosis muy masticables. En el mundo abierto en el que estamos, estamos acostumbrados cada vez más al contexto Twitter, de pocos caracteres. Entonces bueno, creo que está bastante bien dosificada esa información y la formación.  (Caregiver 01) | “The simplicity and clarity, the information come in very concise pills. It does not require a super long text that can make you feel tired, the information is very well dosed. … It is distributed in very chewable doses. In the open world we live in, we are more and more used to the Twitter context, with just a few characters. So, I think that this information and training is quite well dosed.”  (Caregiver 01) |
| Result of the Adhera© Caring Digital Program Usage | “Como que es algo perfectamente normal, que no es que le ha pasado esto a mi hija y nació así. Es normal. Es una enfermedad como cualquier otra que tiene remedio… Le daba un poco vergüenza y leyendo conmigo eso… pues “mira, que no debería darme vergüenza que es así. Al contrario, tengo que decir que soy valiente, mira. Que me pincho sola. Y voy a crecer, y no hay ningún problema… Me llevo con ella yo creo que mucho mejor que antes. Como que le da más confianza, no sé cómo explicarlo. Lo aceptamos más.”  (Caregiver 04) | “It's a perfectly normal thing, it's not like this happened to my daughter and she was born like this. It's normal. It's a disease like any other that can be cured. … she was a little embarrassed and [after] reading that with me... well, "look, I shouldn't be ashamed, that's the way it is. On the contrary, I have to say that I am brave, look. That I inject myself. And I'm going to grow up, and there's no problem. … I think I get along with her much better than before. It gives her more confidence, I don't know how to explain it. We accept it more.” (Caregiver 04) |
| Improvement opportunities | Creo que al comienzo te lo he dicho. Me parece una aplicación muy positiva, muy buena. Que a las familias que comienzan creo que les va a ayudar mucho, que les va a clarificar. Y a las familias que llevamos mucho tiempo si se comparte con ellas, también nos va a afianzar en lo que ya estamos haciendo durante años. A los que comienzan efectivamente los va a ayudar muchísimo más. Conforme la iba leyendo iba diciendo “ojalá yo hubiera tenido esto cuando empecé con esto.  (Caregiver 07) | “I think I told you that at the beginning. I think it is a very positive, very good application. I think it will help families who are just starting out a lot, it will clarify things for them. And for families that have been using it for a long time, if it is shared with them, it will also strengthen what we have been doing for years. For those who are just starting out, it will really help them a lot more. As I was reading it I was saying "I wish I had had this when I started with this"” (Caregiver 07) |
|  | Buscaría más alternativas hacia los padres. Está muy bien los premios, está muy bien motivarlos. Pero la motivación muchas veces también viene el hecho de otras alternativas hacia los niños. No se para que ellos mismos también se autopinchen. No sé si buscar otras alternativas, otras fórmulas, otras… no lo se. Porque yo lo busco. Le decía “date cuenta de que si te pincho yo no vas a poder quedarte en ningún lado. Si te pincho yo no vas a poder irte de campamento.” No lo sé, buscar algún… algún cuento motivado a un proceso, así como… un cuento referente a cómo ir a un cumpleaños, cómo comportarse o como relacionarse. No sé si hay algo incomodo como es un pinchazo y haya algún cuento o algo que se pueda leer a los niños durante noches en el cual pueda llegar a que ellos mismos se motiven. No lo sé si en el mercado hay algún cuento. Que seguramente habrá.  (Caregiver 02) | “I would look for more alternatives for the parents. It's very good to award prizes, it's very good to motivate them. But the motivation often also comes from the fact that there are alternatives for the children. I don't know [motivation] for them to start self-injecting. I don't know whether to look for other alternatives, other formulas, other... I don't know. Because I am looking for it, I told him, "be aware that if I inject you, you won't be able to stay anywhere. If I inject you, you won't be able to go camping." I don't know, look for some... some story motivating to [complete]a process, as ... the stories for children in the autism spectrum regarding how to go to a birthday, how to behave or socialize. I don't know if there is something uncomfortable like an injection, and there is some story or something that can be read to the children during nights that they can motivate themselves with. I don't know if there are any stories on the market. There surely will be”  (Caregiver 02) |
|  | Quitar yo creo nada. Añadir lo que te comentaba, la aparte de esas dos pequeñas prácticas que hay de meditación, de respiración. Si hubiera alguna más estaría bien. Ya no se si podría haber algún tipo de compartir experiencias entre personas que estamos entre papás, entre niños. Seguramente hay recursos que entre unos y otros… o situaciones cómo manejarlas. No sé si es factible en la aplicación o no.  (Caregiver 06) | “I don't think anything should be removed. Add what I was telling you, apart from those two small meditation and breathing practices. If there were any more it would be good. I don't know if there could be some kind of sharing of experiences among people who are among parents, among children. Surely there are resources that we can use with each other... or situations how to handle them. I don't know if it is feasible in the application or not.” (Caregiver 06) |
| Contents: Managing GDH | Esa información nos falta al principio y crea esa incertidumbre. Pensar que le ponemos y que efectos tendrá.  (Caregiver 08) | “This information is missing at the beginning and creates uncertainty. Thinking about what we put in it and what effects it will have.” (Caregiver 08) |
|  | Que si poner un hielo, que si hablar de otra cosa. No se típicos consejos que da para mitigar lo que es el momento del pinchazo o información de la que da. Por ejemplo, leía por ejemplo que a veces les causa dolor y otras veces no. Es algo que hemos experimentado nosotros y no se sabe el motivo. A mí es algo que me llama la atención. Nunca sabía si era porque el niño se quejaba porque se quejaba o porque verdaderamente le duele o porque la aguja verdaderamente es muy fina. Por eso, tener esa constancia que a veces duele y no se sabe porque duele y si no duele no se enteran.  (Caregiver 07) | “(Talking about benefits of using the solution). Using ice or talking about something else... the typical advice given to mitigate the pain perception due to injections or the information given. For example, I read that sometimes it causes pain and other times it does not. It is something that we have experienced, and it is not known why. To me, it is something that catches my attention. I never knew if it was because the child was complaining because it really hurts, or because the needle is really very fine. So, I think having that knowledge regarding sometimes it hurts but you don't know why and sometimes it doesn't hurt is really important.” (Caregiver 07) |
| Contents: health habits to improve dealing with GHD | Bueno menos útiles, no porque no sean útiles. Quizás por ejemplo en el tema de la alimentación. Que es información muy general, pero está muy bien que se recuerde porque también no en todas las familias tienes el mismo pensamiento en relación a la alimentación o la importancia de una alimentación sana. Pero quizás es la parte, si tengo que decir menos útil, esa.  (Caregiver 07) | “Well, less useful, but not because they are not useful. Perhaps, for example, the component focused on healthy eating. It is very general information, but remembering it is important because not all families have the same opinion regarding diet or the importance of healthy eating. But maybe that's the less useful part, if I have to say one.” (Caregiver 07) |
| Contents: Adjusting to living with a GHD | En mi caso, el primer apartado que tiene que ver con el trastorno. Puedo recordar cosas que quizás se habrían olvidado  (Caregiver 06) | “In my case, the first section related to the disorder. I can remember things that perhaps would have been forgotten” (Caregiver 06) |
| Mental wellbeing exercises | A ver... Me llevo con ella yo creo que mucho mejor que antes. Porque es como que “mama, mira lo que puedo hacer”. Como que le da más confianza, no sé cómo explicarlo. Lo  aceptamos más. Es nuestro momento. Incluso me dijo anteayer “yo cuando no haga más las sesiones me parecerá extraño mama. Ese era nuestro momento” Yo creo que afecta mucho, se habla de muchas cosas, y como lo leímos juntas…lo comentamos.  (Caregiver 04) | “... I think I get along with her much better than before ... "mom, look what I can do". It gives her more confidence, I don't know how to explain it. ... It's our moment. She even told me the day before yesterday "when I don't do the sessions anymore it will seem strange to me, mom. That was our moment" I think it affects a lot, we talk about many things, and since we read it together (talking about the educational content) ... we commented on it.” (Caregiver 04) |
|  | A mi sobre todo me ha gustado todo lo que es la práctica de meditación. Y te diría, incluiría un poco más. De cuidadores. Toda esa vertiente de trabajar un poco como apoyo como cuidador, eso me ha gustado.  (Caregiver 03) | “I especially liked the meditation practice. And I would tell you, I would include a little bit more. Regarding caregivers, I liked all contents focused on working as a support, as a caregiver.” (Caregiver 03) |
